# Systematic review and meta-analysis of the value of initial biomarkers in predicting adverse outcome in febrile neutropenic episodes in children and young people with cancer

**DOI:** 10.1186/1741-7015-10-6

**Published:** 2012-01-18

**Authors:** Robert S Phillips, Ros Wade, Thomas Lehrnbecher, Lesley A Stewart, Alex J Sutton

**Affiliations:** 1 Center for Reviews and Dissemination, University of York, York, UK; 2 Department of Paediatric Haematology and Oncology, University of Frankfurt, Frankfurt, Germany; 3 Department of Health Sciences, University of Leicester, Leicester, UK

## Abstract

**Background:**

Febrile neutropenia is a frequently occurring and occasionally life-threatening complication of treatment for childhood cancer. Many biomarkers have been proposed as predictors of adverse events. We aimed to undertake a systematic review and meta-analysis to summarize evidence on the discriminatory ability of initial serum biomarkers of febrile neutropenic episodes in children and young people.

**Methods:**

This review was conducted in accordance with the Center for Reviews and Dissemination Methods, using three random effects models to undertake meta-analysis. It was registered with the HTA Registry of systematic reviews, CRD32009100485.

**Results:**

We found that 25 studies exploring 14 different biomarkers were assessed in 3,585 episodes of febrile neutropenia. C-reactive protein (CRP), pro-calcitonin (PCT), and interleukin-6 (IL6) were subject to quantitative meta-analysis, and revealed huge inconsistencies and heterogeneity in the studies included in this review. Only CRP has been evaluated in assessing its value over the predictive value of simple clinical decision rules.

**Conclusions:**

The limited data available describing the predictive value of biomarkers in the setting of pediatric febrile neutropenia mean firm conclusions cannot yet be reached, although the use of IL6, IL8 and procalcitonin warrant further study.

## Background

With multi-modality therapies, children with malignancy have an excellent chance of survival, with overall rates approaching 75% [1]. Deaths are largely due to their disease, but around 16% of deaths are from complications of therapy [2,3]. This proportion depends on the underlying malignancy, and the risk of death from infection remains high in some groups, for example, acute myeloid leukemia [4]. Robust risk stratification, which reliably predicted those children at high risk of complications, could target more aggressive management, where children at very low risk of having a significant infection could be treated with reduced intensity and/or duration of hospitalized antibiotic therapy [5]. There are a wide range of differing approaches to this risk stratification, largely built on simple clinical data [6-8], demonstrating only moderate discriminatory ability.

The ability of specific serum biomarkers to predict adverse consequences in patients with febrile neutropenia has been explored, for example, C-reactive protein (CRP), pro-calcitonin (PCT), interleukin-6 (IL6) or interleukin-8 (IL8) [9-12]. These studies have been small in the numbers of patients and episodes and the researchers could not reach definitive conclusions. Drawing these reports together and synthesizing their results should improve our understanding of their clinical usefulness.

Although systematic reviews have been conducted previously in adults [13] and non- immunocompromised children [14,15], their results are difficult to compare. There are data to suggest children and adults with neutropenic fever vary in the nature of the infections which afflict them [16], implying any review needs to take into account the specific population under study.

This review aimed to identify, critically appraise and synthesize information on the use of biomarkers at initial evaluation for the prediction of the outcome of febrile neutropenic episodes in children/young adults and to highlight important problems in the current methods used in such analyses.

## Methods

The review was conducted in accordance with "Systematic reviews: CRD's guidance for undertaking reviews in health care" [17] and registered on the HTA Registry of Systematic Reviews: CRD32009100485. It sought studies which evaluated the diagnostic ability of serum biomarkers of inflammation/infection in children or young people aged 0 to 18 years of age, taken at the onset (within 12 hours) of an episode of febrile neutropenia. Both prospective and retrospective cohorts were included, but those using a case-control approach were excluded as these have been previously shown to exaggerate diagnostic accuracy estimates [18].

### Search strategy and selection criteria

An electronic search strategy (See Additional file 1) was developed to examine a range of databases from inception to February 2009, including MEDLINE, EMBASE, CINAHL, Cochrane Database of Systematic Reviews, Database of Abstracts of Reviews of Effects, Health Technology Assessment Database, Cochrane Central Register of Controlled Trials, Conference Proceedings Citation Index - Science and LILACS.

Reference lists of relevant systematic reviews and included articles were reviewed for further relevant articles. Published and unpublished studies were sought without language restrictions. Non-English language studies were translated. Two reviewers independently screened the titles and abstracts of studies for inclusion, and then the full text of retrieved articles. Disagreements were resolved by consensus.

The validity of each study was assessed using 11 of the 14 questions from the Quality Assessment of Diagnostic Accuracy Studies (QUADAS) assessment tool for diagnostic accuracy studies [19] (see footnote of Additional file 2). The QUADAS tool was adapted specifically for the review, as suggested by current guidance [20], omitting questions on "time between index and reference test", "intermediate results" and "explanation of withdrawals". The index test (biomarkers) and reference test were always examined within a single episode of febrile neutropenia, making this question indiscriminating. Tests of biomarkers are not reported as 'positive' and 'negative', and so "intermediate" results are not found in these types of studies. Rather than addressing "incomplete data" as a validity item, it was addressed in the data analysis.

Data were extracted by one researcher using a standardized data extraction form and accuracy confirmed independently by a second; except with foreign language papers where a translator working with a reviewer undertook the extraction. Clinical data extracted included participant demographics, geographical location, participant inclusion/exclusion criteria and antibiotics used. Methodological information included methods used to adjust the predictive estimate, including the variables considered, and methods of analysis. The reference standard outcomes considered relevant included survival, need for intensive/high-dependency care, single organ impairment, invasive bacterial or fungal infection, presence of documented infection, including radiologically confirmed pneumonia, and duration of hospitalization. The sensitivity and specificity of the biomarkers were extracted, preferentially as 2 × 2 tables comparing dichotomized test results against the reference standard. Where data were only presented as mean and standard deviation, conversion was undertaken using the assumption of Normality and deriving a 2 × 2 table for cut-offs reported by other studies (Anzures, Cochrane Colloquium Freiburg 2008).

### Methods of analysis/synthesis

Quantitative synthesis was undertaken for studies which tested the same diagnostic test for similar clinical outcomes and, where appropriate, was investigated for sources of heterogeneity.

Three approaches were used for meta-analysis. The first approach (Method 1) pooled data from the most commonly reported threshold, using a single data point from each study that provided relevant information, for example, each study reporting serum CRP > 50 mg/dL. This was expressed as the average test sensitivity and specificity, with a 95% confidence interval. This was calculated by fitting the standard bivariate random effects model using STATA (version 10) [21] with metandi [22] and midas [23] for analyses of four or more studies; for those with fewer than four studies a random effects linear regression was directly fitted using xmelogit. The bivariate model is the most commonly used technique in diagnostic meta-analysis, and has benefits of being easily interpretable, as it provides a point estimate of the test accuracy in this context for a defined cut-off value, and is technically straightforward to undertake. Its weaknesses lie in the partial use of data from all the included studies, (since accuracy at multiple test cut-offs was available from many studies), which may lead to reduced power and consequent imprecision, and increased risk of bias from a selective use of data.

The second approach (Method 2) again pooled one data point from each study, but combined information from multiple thresholds, for example, serum CRP > 40 mg/dL, > 50 mg/dL and > 90 mg/dL, and the output was expressed as a hierarchical summary receiver operator curve (HSROC). The HSROC describes the relationship between sensitivity and specificity derived from the individual receiver operator curves (ROC) of each study. In this way, it describes the 'average' relationship between a continuous cut-off value and discriminatory ability in the 'average' population. This increases the information used in the meta-analysis and better represents the data. The same routines were used in STATA (version 10) [21] to produce these estimates. This approach is again technically straightforward to perform, and the output allows clinicians to estimate how changing thresholds will alter the diagnostic utility of the test under study. Its weaknesses relate to the difficulty in interpreting exactly what performance is associated with each cut-off level, and its lack of explicit inclusion of threshold data when producing the curve.

The third analysis (Method 3) allowed multiple data points from multiple thresholds from each study to be included, and was undertaken using a multinomial random effects method deriving proportions of the population with/without the outcome at each cut-off level of the biomarkers. These were then used to derive likelihood ratios for each level [24]. This provides the richest model, including all of the available data from the studies and should produce the clearest possible descriptions of the predictive value of the biomarkers. This was accomplished using a previously published method [8] and non-informative priors. Analyses were undertaken using WinBUGS 1.4.3 [25]. The code is available upon request. This method is theoretically superior to the other methods, as it includes all of the available data, unlike Method 1, explicitly uses the threshold values, unlike Method 2, and produces threshold-specific estimates of diagnostic test performance, which can be interpreted directly by clinicians. It is the most technically challenging of all the methods used, requiring specific code to be written for each analysis, rather than the use of easily available software packages.

Heterogeneity between study results was explored through consideration of study populations, design, predictor variables and outcomes. Meta-regression was not undertaken due to the small number of studies. When quantitative synthesis was not possible, a narrative approach was used to synthesize the information.

## Results

**Figure 1:**
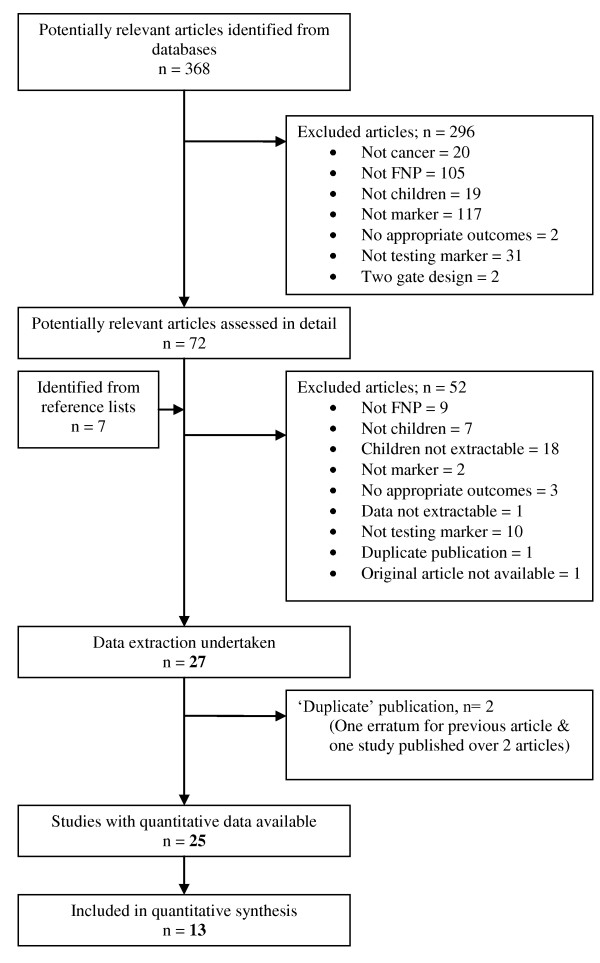
**Flow diagram of study selection process**.

Three hundred, sixty-eight articles were initially reviewed, and 72 retrieved for more detailed examination. Twenty-five articles provided quantitative outcome data in the form required for the review (see Additional file 3). The included studies included 2,089 patients and over 3,585 episodes, assessing 14 different markers of inflammation or infection (see Table 1). The study outcomes were grouped into: bacteremia, invasive fungal infection, significant/documented bacterial infection, sepsis and death. The population in the studies varied, with most being a mixture of hematological and solid malignancies, and very little data from stem cell transplant recipients (see Table 2 for further detail). Thirteen of these contributed to 1 or more meta-analyses while the remaining 12 studies did not provide data which could be included in any meta-analysis. (see Figure 1). Three biomarkers and 2 outcomes could be included in the meta-analysis: 11 studies provided data on CRP [9,26-35] and documented infection. Four studies provided data on PCT [28,29,31,33] and documented infection. Four provided data on IL6 [31,36-38] and documented infection or gram negative bacteremia.

**Table 1:** Summary of biomarkers reported across all included studies

Biomarker	Total studies
C reactive protein	20
Interleukin 6	10
Interleukin 8	10
Procalcitonin	8
Tumor necrosis factor α	2
Interleukin 10	1
Monocyte chemoattractant protein-1	1
Erythrocyte sedimentation rate	1
Adenosine deaminase	1
Serum Amyloid A Protein	1
Interleukin 1	1
Interleukin 5	1
Interleukin 12	1

Interleukin 2 - receptor	1

**Table 2:** Details of biomarkers, patients and endpoints in 25 included studies

Citation	Underlying conditions	Underlying conditions	Markers studied	Number of patients	Number of episodes	Endpoints studied	Comments on endpoints
Ammann 2003	Pre-B-cell ALL = 94, Other diagnosis = 191		CRP	111	285	Significant bacterial infection	Defined as death from bacterial infection, a positive culture of normally sterile body fluids, radiologically proven pneumonia, clinically unequivocal diagnosis of a bacterial infection, or a serum CRP above 150 mg/L as an indirect sign suggesting severe bacterial infection

Barnes 2002	No data given		PCT	37	39	Length of stay	Stay of < 5d or ≥5d

de Bont 1999	ALL = 8, AML = 20, CML = 2, Lymphoma = 16, Solid tumor = 7		CRP, IL6, IL8	19	72	Bacteremia	

Diepold 2008	ALL = 21, AML = 1, JMML = 1, Relapsed AML after SCT = 1, Solid tumor = 39, Hematological disorder after SCT = 4, Hematological disorder without SCT = 1		CRP, IL6, IL8	69	123	Bacteremia Fever lasting ≥5d but culture -ve	

Dylewska 2005 a and b	No data given		CRP, PCT	66	108	Bacteremia Clinically defined infections (UTI, neurological, GI or respiratory) Microbiologically defined other infection	FUO was the default category

El-Maghraby 2007	ALL = 37, AML = 39, Lymphoma = 39		CRP, IL8, MCP	76	85	Bacteremia or clinically documented infection	

Hatzistilianou 2007	All patients had ALL		CRP, PCT	29	94	Microbiological or clinically documented infection (excludes viral)	

Heney 1992	ALL = 17, AML = 10, Lymphoma = 2, Solid tumor = 18		CRP, IL6	33	47	Bacteremia	

Hitoglou-Hatzi 2005	All patients had ALL		CRP, PCT, tADA	67	Not stated	Significant bacterial infection	

Hodge 2006	No overall data given		IL8, IL5	31	31	Positive blood culture	

Katz 1992	Haematological malignancy = 82, Solid tumor = 40		CRP	74	122	Clinically or bacteriologically documented infection Septicemia (+ve blood cultures and unwell clinical appearance)	

Kitanovski 2006	Hematological malignancy = 50, Solid tumor = 18		CRP, PCT, IL6	32	68	Bacteremia and clinical sepsis Clinically or microbiologically documented local infection	

Lehrnbecher 1999	ALL = 17, AML = 7, Lymphoma = 5, Solid tumor = 27		CRP, PCT, IL6	56	121	Clinically documented infection Fungal infection Bacteremia (gram-type)	FUO was the default category

Lehrnbecher 2004	ALL = 48, AML = 15, Lymphoma = 16, Histiocytosis = 1, Solid tumor = 66		IL6, IL8	146	311	Significant bacterial infection	Defined as bacteremia, localised infection or pneumonia

Riikonen 1992	No data given		IL6, IL1, TNF, SAA	46	105	Bacteremia suspected sepsis Focal infection	"No infection" was the default category

Riikonen 1993	ALL = 20, AML = 24, Solid tumor = 47		CRP	46	91	Bacteremia suspected sepsis Focal infection	"No infection" was the default category

Santolaya 1994	Leukemia = 47, Lymphoma = 17, Solid tumor = 11		CRP	75	85	Documented bacterial infection Probable bacterial infection Viral infection	Documented bacterial infection defined as bacteremia (two sets positive for commensals) or sterile site infection; Probable bacterial infection defined as cultures negative but severe medical course, for example, purulent gingivostomatitis, CXR+; FUO was the default category

Santolaya 2001	ALL = 40%, AML = 8%, Relapsed leukemia = 14%, Lymphoma = 6%, Sarcoma = 17%, Other solid tumor = 15%		CRP	257	447	Invasive bacterial infection	Defined as positive blood cultures - 2 for CoNS, positive bacterial culture from usually sterile site, or sepsis syndrome and/or focal organ involvement and haemodynamic instability and severe malaise

Santolaya 2002	ALL = 92, AML = 14, Lymphoma = 10, Solid tumor = 54		CRP	170	263	Invasive bacterial infection	Defined as positive blood cultures - 2 for CoNS, positive bacterial culture from usually sterile site, or sepsis syndrome and/or focal organ involvement and haemodynamic instability and severe malaise

Santolaya 2007	No overall data given		CRP	219	373	Death	

Santolaya 2008	No overall data given on diagnoses.		CRP, PCT, IL8	278	566	Severe sepsis	Defined as sepsis + respiratory or cardiac compromise, or + 2 other-organ compromise) not apparent during the first 24 h of admission

Secmeer 2007	ALL and AML = 9, Lymphoma = 14, Sarcoma = 7, Histiocytosis = 1, Other solid tumor = 18		CRP, PCR, ESR	49	60	Bacteremia Documented bacterial infection (microbiologically or clinically) Duration of fever	

Soker 2001	ALL = 17, AML = 4, Lymphoma = 2		IL6, IL8, IL2R, IL1, TNF-alpha	23	48	Bacteremia	

Spasova 2005	ALL = 23, AML = 1, Lymphoma = 6, Solid tumor = 11		CRP, IL6, IL8, IL10	24	41	Bacteremia Microbiologically or clinically proven local infections without bacteremia	

Stryjewski 2005	ALL = 35, AML = 2, Sarcoma = 8, Other solid tumor = 11		PCT, IL6, IL8	56	Not stated	Sepsis Septic shock	Sepsis (positive culture - two consecutive +ve if CoNS, fever, tachycardia, or tachypnoea); septic shock defined as sepsis plus need for inotropes/vasopressors

CRP, C-reactive protein; CoNS, coagulase negative staphylococcus; ESR, erythrocyte sedimentation rate; FUO, fever of unknown origin; IL, interleukin; MCP, monocyte chemoattractant protein; PCT, pro-calcitonin; SAA, serum amyloid protein A; tADA, t- Adenosine Deaminase; TNF, Tumor necrosis factor

### Quality assessment

The studies varied in quality; see Additional file 2. The major deficiencies in most studies were in a failure to report if the marker test and outcomes were interpreted blind to each other. One study [26] assessing CRP demonstrated a potential contamination of the reference standard with the diagnostic test: the outcome included CRP > 150 mg/dl. One short report did not detail the exact outcome used [39]. Twenty different definitions of 'febrile neutropenia' were described, including six definitions of neutropenia ranging from < 200 cells/mm^3 ^to < 1,000 cells/mm^3^; four definitions of peak fever, from > 37.5°C to > 39°C; and six of sustained temperature, from > 38°C to > 38.5°C over varying durations. There were a total of 14 combinations to define 'febrile'.

### Data handling and analysis

Detailed analysis of the statistical modeling used in the original studies revealed potential problems in adjustment of estimates for other factors, limited event-per-variable ratios, poorly described handling of multiple episodes and missing data, and use of data-driven dichotomies in the reporting of test accuracy.

### Diagnostic test performance

Data were available for meta-analysis for CRP for microbiologically or clinically documented infection; for PCT assessing microbiologically or clinically documented infection; and IL6 reporting microbiologically or clinically documented infection, and gram negative bacteremia. Individual results for these studies and outcomes are given in Additional file 3.

Meta-analysis using the three specified approaches illustrated how the standard, simple approach to pooling of test accuracy data may be misleading and lead to clinically inappropriate conclusions.

**Figure 2:**
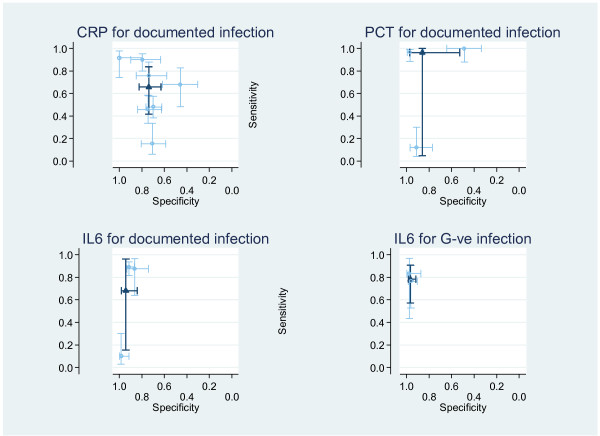
**Method 1: bivariate pooled estimates of sensitivity and specificity for CRP, PCT and IL6**. The plots indicate individual study estimates of sensitivity and specificity with 95% confidence intervals demonstrated by dashed lines, the solid lines indicate the meta-analysis result.

For studies with similar outcomes and where identical cut-off values were reported in more than one study, meta-analysis was undertaken to calculate a single diagnostic test accuracy estimate using the standard random effects bivariate approach: Method 1 (see Table 3 and Figure 2). This approach is the commonly applied technique, yet does not take into account the inconsistency of the full data set (see Methods).

**Table 3:** Bivariate estimates of diagnostic precision of various biomarkers and outcomes

Marker	Outcome	Cut-off	Sensitivity (95% CI)	Specificity (95% CI)
CRP (7 studies, 731 episodes)	Documented infection	> 50 mg/dl	0.65 (0.41 to 0.84)	0.73 (0.63 to 0.82)

PCT (3 studies, 216 episodes)	Documented infection	> 0.2 mg/ml	0.96 (0.05 to 0.99)	0.85 (0.53 to 0.97)

IL6 (3 studies, 457 episodes)	Documented infection	> 235 pg/ml	0.68 (0.15 to 0.96)	0.94 (0.84 to 0.98)

IL6 (2 studies, 166 episodes)	Gram negative bacteremia	> 1,000 pg/ml	0.78 (0.57 to 0.91)	0.96 (0.92 to 0.99)

There is marked heterogeneity in the results of this meta-analysis, with sensitivity heterogeneous in all markers, and specificity most heterogeneous in PCT and CRP. This can be appreciated by comparison of the point estimates and confidence intervals in the y (sensitivity) axis and x (reverse-specificity) axis in Figure 2.

**Figure 3:**
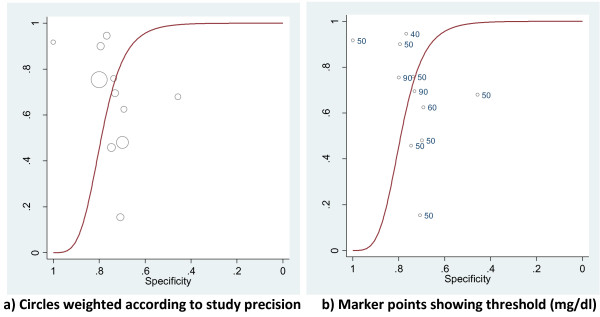
**Method 2: hierarchical summary receiver operator curve plots of CRP for the diagnosis of documented infection**. **a) **Circles weighted according to study precision **b) **Marker points showing threshold (mg/dl).

Using the second approach, producing HSROC, it was possible for CRP and PCT to detect 'documented infection': Method 2. No further HSROC curves were derived as no other combinations of outcome and biomarker were available in more than three studies. In this analysis, the threshold variation was not adhered to, as can be seen in the example of CRP. Figure 3a shows the curve without threshold, and 3b shows how the values are not in the order expected. The expectation is that a higher cut-off produces a lower sensitivity and higher specificity; this is not the case and so this makes clinical interpretation of the curve impossible.

**Figure 4:**
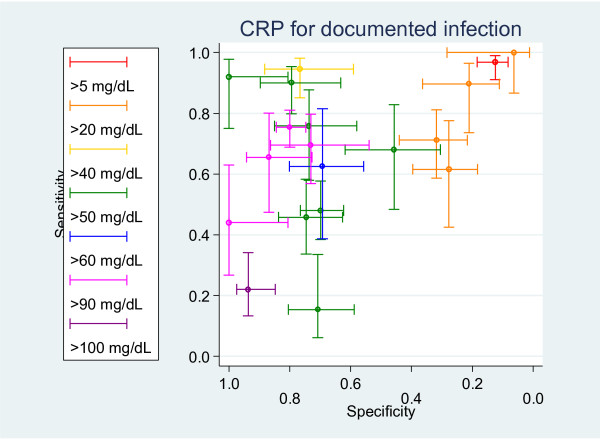
**Method 3: ROC space plot of CRP for documented infection (all thresholds)**.

The meta-analysis method (Method 3), which maximizes the use data, including multiple thresholds from studies using a multinomial random effects model, demonstrates that these problems arise because of the inconsistencies in the reported data. Again, the CRP data are used to demonstrate this (see Figure 4). This shows that some of the lower thresholds are less sensitive than higher thresholds; for example, using a cut-off of > 20 mg/dL produced more false negative results than a cut-off of > 50 mg/dL. These differences are beyond those expected by chance and led to the analyses producing clinically meaningless results. This is likely to be due to the extreme heterogeneity and sparse data.

Data on the diagnostic value of nine other markers are presented in Table 4. IL8 was most frequently described [27,38,39]. Most of these studies were exploratory, proposing new biomarkers and deriving cut-offs, for example, Monocyte chemoattractant protein-1 or Adenosine deaminase. The predictive value of these biomarkers is also heterogeneous, and subject to potential biases.

**Table 4:** Estimates of diagnostic precision of various markers and outcomes in single studies.

Citation	Marker and Cutpoint	Outcome	Sensitivity (95% CI)	Specificity 95% CI)
Santolaya 2008	IL8 200	Sepsis	0.49 (0.4 to 0.58)	0.71 (0.67 to 0.75)

Diepold 2008	IL8 30	Prolonged illness	0.87 (0.78 to 0.93)	0.61 (0.42 to 0.76)

Diepold 2008	IL8 90	Bacterial infection	0.64 (0.39 to 0.84)	0.62 (0.52 to 0.71)

El-Maghraby 2007	IL8 62	Documented infection	0.71 (0.59 to 0.81)	0.77 (0.58 to 0.89)

Lehrnbecher 2004	IL8 320	Documented infection	0.56 (0.46 to 0.65)	0.79 (0.73 to 0.84)

Lehrnbecher 2004	IL8 500	Documented infection	0.44 (0.35 to 0.54)	0.89 (0.84 to 0.93)

El-Maghraby 2007	MCP 350	Documented infection	0.64 (0.52 to 0.75)	0.92 (0.76 to 0.98)

Hitoglou-Hatzi 2005	tADA 35 U/l	Significant bacterial infection	1.0 (0.88 to 1.0)	1.0 (0.91 to 1.0)

Riikonen 1992	TNF 40	Bacteremia or focal infection	1.0 (0.88 to 1.0)	0.07 (0.03 to 0.15)

Hodge 2006	IL5 17	Positive blood culture	0.5 (0.22 to 0.79)	Could not calculate

Hodge 2006	IL5 and 8 combined > 17 and > 220	Positive blood culture	1.0 (0.68 to 1.0)	0.87 (0.68 to 0.96)

Soker 2001	IL-2R	Bacteremia	Median (range) 5,230 U/mL (1,120 to 7,600)	1,190 (724 to 5,400)

Soker 2001	TNF-alpha	Bacteremia	8.4 (4.0 to 68.2)	7.8 (3.0 to 37.2)

Secmeer 2007	ESR	Bacteremia	"not statistically significantly different between patients with and without documented infection"	

## Discussion

This systematic review of the predictive value of serum markers of inflammation and infection in children presenting with febrile neutropenia found 25 studies reporting 14 different markers. Of these, CRP, PCT, IL6 and IL8 were most commonly examined. The finding of a diverse range of potentially useful markers, but such little consistency across studies, is unfortunately common in such research [40], and may reflect the relative lack of coordination in supportive care studies.

The studies presented similar challenges in reporting, methodology and analysis. Reporting if the test was interpreted 'blind' to the results of the outcome analysis, and *vice-versa*, was very poorly reported. Many studies failed to assess if the marker had supplementary value above the simple admission data collected by clinicians at every encounter: age, malignancy, temperature, vital statistics and blood count. Analysis of the data was frequently undertaken by episode, with no account of multiple admissions for the same patient. Such an analysis ignores the variation which may be expected from genetic polymorphisms for the production of the biomarker under investigation [39], or in individual genetic susceptibility to infection [41,42]. The biomarker cut-off values reported were frequently derived from the dataset to which they were then applied, which is likely to produce significant overestimations of accuracy [43]. The data were sometimes presented as mean and standard deviation estimates, from which measures of test accuracy were derived. Although this may raise concerns because of the assumption of a normal distribution, there is some empiric justification for this procedure [44].

Quantitative meta-analysis using three approaches demonstrated how the commonly used, simple techniques may fail to reflect inconsistencies in the whole data set and so produce misleadingly precise results. The example of this review is important to recall when appraising other reviews where inconsistencies may not have been as extensively investigated.

The analysis undertaken using only the most commonly reported cut-off in a restricted number of studies produced excessively precise results which did not reflect the uncertainty of the whole data set, and so should be rejected. A similar problem was found with the use of data points with different thresholds to produce a hierarchical summary receiver operator curve (HSROC). The HSROC modelled by these techniques does not take into account the actual value of the thresholds. This is frequently reasonable: it is impossible to quantify the thresholds used by different radiologists to call a radiograph 'positive' for pneumonia. In cases where the values are known, an ordered relationship should be possible to determine, flowing from high to low cut-offs from left to right on the curve. This ordered relationship did not hold true for analyses of CRP and PCT and so should call into question analyses in other studies which do not assess whether thresholds vary according to the implicit structure of the model.

A previously developed [8] technique to undertake the ordered pooling of all the results was used to attempt to overcome these difficulties of only selective use of the data, and of incorrect relationships between test thresholds. This approach failed to produce meaningful results for the ability of PCT and CRP to identify patients who developed a documented infection, reflecting the inconsistencies and great heterogeneity of the data.

Some of the observed heterogeneity may be due to differences in measurement between apparently similar outcomes. While bacteremia is likely to be similarly reported across the studies, the diagnosis of a soft-tissue infection may vary between clinicians and centers. Very few studies reported in detail the exact definitions of the outcomes they reported. Further variation may have been introduced by the varying definitions of fever and neutropenia. In this review, 20 different combinations of criteria were used to define febrile neutropenia. These data could not be directly assessed to explore their relationship with the diagnostic value of the biomarkers, but as the depth of neutropenia and peak, and duration of temperature may affect the generation of biomarkers, the variation may further account for some of the heterogeneity. Additionally, although the assay techniques used in the studies were reported to be similar, there was no calibration of assays across the various studies. Other differences in the populations studied, such as the nature of the malignancies, recent surgical interventions and duration of therapy, may also add heterogeneity to interpreting markers which are themselves affected by a malignant disease. A more prosaic reason for heterogeneity may be publication bias: the tendency for reports demonstrating good predictive value to be published than those showing poor discrimination [45-47].

In order to interpret the information from this review in a clinically meaningful way, both the estimates of predictive effectiveness and the uncertainty that surrounds these estimates need to be taken into account. CRP has been most extensively studied in this setting; it is a ubiquitous test and the only one which has been shown to add to the predictive ability of clinically-based decision rules [26,34]. These studies chose two differing cut-offs (> 50 mg/dl [26] or > 90 mg/dl [34]). It is at best only moderately discriminatory in the setting of detecting documented infection (Sensitivity 0.65; 95% CI 0.41 to 0.84, Specificity 0.73; 95% CI 0.63 to 0.82), which is in keeping with estimates drawn from its value in the detection of serious bacterial infection in non-neutropenic children [48], and may be a significant overestimation of its value. The clinical role of CRP as a screening tool may be limited, however, if another biomarker is shown to be a more discriminatory test.

Data from this review and meta-analytic comparisons of CRP and PCT in the non-neutropenic population [49] are suggestive of the improved predictive value of PCT over CRP. This has a strong pathophysiological basis, as PCT levels are reported to rise within 3 to 4 hours in response to infection as compared with the 24 to 48 hours required for CRP [33]. However, the data for the improved predictive value of PCT are quite varied (see Additional file 3 and previously published reviews [13]). This may be related to the degree of neutropenia, as reports from the post-transplant setting have shown disappointingly poor discrimination [50], or this again may be due to small studies and publication bias [47,51]. Based on the data from this review, procalcitonin cannot yet be recommended for use in routine clinical practice

Similar pathophysiological claims for improved predictive ability can be advanced for IL6 and IL8 [52]. In this review, IL6 level shows potential to be a better discriminator than CRP of those children who will develop a serious infectious complication. IL8 also appears to have moderate discriminatory ability and has been used in combination with clinical data in a small pilot study to withhold antibiotics to a highly select group of patients with febrile neutropenia [53]. Both of these cytokines show promise, and should be subject to further investigation.

Given the very limited data available for other potential biomarkers of infection in the setting of pediatric febrile neutropenia identified by this review, no strong clinical conclusions for their use can be reached without further studies.

These conclusions are drawn from an extensive and detailed systematic review of the available evidence using advanced techniques of meta-analysis, supplemented by rational clinical and pathophysiological reasoning. It should be clearly understood that they are uncertain and unstable, as only small amounts of new data may substantially alter these findings.

## Conclusions

This review demonstrates flaws in our current understanding of the value of biomarkers in the prediction of adverse outcomes from episodes of febrile neutropenia, but also provides us with clear opportunities for development. All further investigation should estimate the additional value of biomarker measurements, beyond the discrimination already achieved by clinical variables. This should take into account key features of the treatment, for example, stem-cell transplantation and any clinically defined risk stratification already undertaken.

This includes the use of individual patient data (IPD) meta-analysis, which should allow the effective added-value of markers to be measured when the best clinical data have been taken into account in differing sub-groups. Such a venture is in progress [54]. The biomarkers IL6, IL8 and PCT appear promising, and should certainly be subject to new primary studies investigating more thoroughly the prediction of significant infectious morbidity, which includes both clearly defined infections and the sepsis syndrome, across a variety of clinical settings. By developing harmonized definitions of outcomes for such studies, greater confidence could be placed upon their results. The new SIOP Supportive Care group is ideally placed to lead on such a venture, and allow pediatric oncology/hematology to once more push the boundaries of international, collaborative clinical research.

## Abbreviations

CRD: Centre for Reviews and Dissemination; CRP: C-reactive protein; FNP: febrile neutropenia; HSROC: hierarchical summary receiver operator curve; IL6: interleukin 6; IL8: interleukin 8; PCT: procalcitonin; QUADAS: Quality Assessment of Diagnostic Accuracy Studies; ROC: receiver operator curve; SIOP: Societe Internationale d'Oncologie Paediatrique.

## Competing interests

The authors declare that they have no competing interests.

## Authors' contributions

RSP developed the concept for the review with LS and AJS. The protocol was further developed by RSP, RW, LS and AJS. Study screening and data extraction were undertaken by RSP and RW. Data analysis was undertaken by RSP and AJS. Interpretation and conclusions were drawn from this work by all authors, with particular clinical input from TL and methodological discussion with AJS. All authors have contributed to and reviewed the final paper. RSP is the guarantor of this work.

## Pre-publication history

The pre-publication history for this paper can be accessed here:

http://www.biomedcentral.com/1741-7015/10/6/prepub

## Supplementary Material

Additional file 1 **Search Strategy (Medline)**. The full search strategy used for the Medline database. Click here for file

Additional file 2 **QUADAS assessments**. Full list of assessed QUADAS (diagnostic test accuracy critical appraisal) criteria for the 25 included studies. Click here for file

Additional file 3 **Per-study results used in meta-analysis**. Individual study results used in pooled analyses presented by marker, outcome and cut-off. Click here for file
