# Supplementary material for: Systematic review and meta-analysis of the value of initial biomarkers in predicting adverse outcome in febrile neutropenic episodes in children and young people with cancer
Source: BMC Med. 2012 Jan 18;10:6. doi: 10.1186/1741-7015-10-6 (PMC3331823; doi:10.1186/1741-7015-10-6)
Supplement: Additional file 1 — Search Strategy (Medline). The full search strategy used for the Medline database. [file 1741-7015-10-6-S1.DOCX]

**Additional File 1. Search Strategy (Medline)**

Example based on OVID-Medline: was adapted for other databases

*FNP identification*

1 Neutropenia/

2 (neutropenia or neutropenic).ti,ab.

3 1 or 2

4 Fever/

5 (fever$ or febril$).ti,ab.

6 4 or 5

7 3 and 6

*Child identification*

8 adolescent/ or child/ or child, preschool/ or infant/ or infant, newborn/ or Puberty/

9 schools/ or schools, nursery/

10 (infan$ or newborn$ or new born$ or baby$ or babies or neonat$ or neonat$ or child$ or schoolchild$ or kid or kids or toddler$ or adoles$ or teen$ or boy$ or girl$ or minor$ or underage$ or under age$ or juvenil$ or youth$ or kindergar$ or nursery or puber$ or prepuber$ or pre puber$ or pubescen$ or prepubescen$ or pre pubescen$ or pediatric$ or paediatric$ or peadiatric$ or school or schools or preschool$ or pre school$ or schoolage$).ti,ab.

11 8 or 9 or 10

*Cancer identification*

12 exp Neoplasms/

13 (cancer$ or neoplas$ or oncolog$ or malignan$ or tumo?r$ or sarcoma$

or leukaemi$ or leukemi$ or chemotherap$).ti,ab.

14 12 or 13

15 14 and 11 and 7

*Markers identification*

1. Biological Markers/
2. (marker$ or serum).ti,ab.
3. (biomarker$ or bio-marker$).ti,ab.
4. or/18-20
5. Cytokines/
6. cytokine$.ti,ab.
7. 22 or 23
8. Interleukin-1/
9. (interleukin-1 or interleukin-i or il-1 or il1).ti,ab.
10. t-helper factor.ti,ab.
11. lymphocyte-activating factor.ti,ab.
12. macrophage cell factor.ti,ab.
13. epidermal cell derived thymocyte-activating factor.ti,ab.
14. or/25-30
15. Interleukin-5/
16. (interleukin-5 or il-5 or il5).ti,ab.
17. eosinophil differentiation factor.ti,ab.
18. t-cell replacing factor.ti,ab.
19. (b-cell growth factor-ii or b-cell growth factor-2).ti,ab.
20. (bcgf-ii or bcgfii or bcgf-2 or bcgf2).ti,ab.
21. or/32-37
22. Interleukin-6/
23. (interleukin-6 or il-6 or il6).ti,ab.
24. plasmacytoma growth factor.ti,ab.
25. b-cell differentiation factor.ti,ab.
26. (b-cell stimulat$ factor-2 or b-cell stimulat$ factor-ii).ti,ab.
27. (bsf-2 or bsf2 or bsf-ii or bsfii).ti,ab.
28. hepatocyte-stimulating factor.ti,ab.
29. hybridoma growth factor.ti,ab.
30. (interferon beta 2 or interferon beta2 or ifn-beta 2 or ifn-beta2).ti,ab.
31. mgi-2.ti,ab.
32. myeloid differentiation-inducing protein.ti,ab.
33. or/39-49
34. Interleukin-8/
35. (interleukin-8 or il-8 or il8).ti,ab.
36. monocyte-derived neutrophil chemotactic factor.ti,ab.
37. neutrophil activation factor.ti,ab.
38. lymphocyte-derived neutrophil-activating peptide.ti,ab.
39. monocyte-derived neutrophil-activating peptide.ti,ab.
40. (alveolar macrophage chemotactic factor-i or amcf-i).ti,ab.
41. anionic neutrophil-activating peptide.ti,ab.
42. cxcl8.ti,ab.
43. macrophage-derived chemotactic factor.ti,ab.
44. neutrophil chemotactic factor.ti,ab.
45. or/51-61
46. Interleukin-10/
47. (interleukin-10 or il-10 or il10).ti,ab.
48. csif-10.ti,ab.
49. or/63-65
50. Interferon-gamma/
51. (interferon-gamma or gamma-interferon or IFN-gamma or IFNgamma).ti,ab.
52. (interferon ii or interferon 2).ti,ab.
53. (type ii interferon or interferon type ii).ti,ab.
54. immune interferon.ti,ab.
55. or/67-71
56. Interferon-beta/
57. (interferon-beta or beta-interferon or IFN-beta or IFNbeta).ti,ab.
58. fibroblast interferon.ti,ab.
59. (interferon-beta1 or beta1 interferon or beta-1 interferon or IFN-beta1 or IFNbeta1).ti,ab.
60. Fiblaferon.ti,ab.
61. or/73-77
62. transforming growth factor beta/
63. (beta transforming growth factor or transforming growth factor beta or tgf-beta or tgfbeta).ti,ab.
64. milk growth factor.ti,ab.
65. platelet transforming growth factor.ti,ab.
66. bone-derived transforming growth factor.ti,ab.
67. or/79-83
68. Antigens, CD70/
69. (CD70 or cd27l or cd27 ligand).ti,ab.
70. 85 or 86
71. Tumor Necrosis Factor-alpha/
72. (tumour necrosis factor or tumor necrosis factor).ti,ab.
73. (tnf or tnfalpha).ti,ab.
74. Cachectin.ti,ab.
75. or/88-91
76. Receptors, Tumor Necrosis Factor, Type II/
77. (tnfrii or tnfr-ii or tnfr2 or tnfr-2).ti,ab.
78. (stnf-ii or stnfrii or stnfr2 or stnfr-2).ti,ab.
79. (tnfr p75 or tnfr p80 or tnf-sr75).ti,ab.
80. (cd-120b or cd120b).ti,ab.
81. tnfrsf1b receptor$.ti,ab.
82. or/93-98
83. C-Reactive Protein/
84. (c-reactive protein or Creactive protein or c-reaction protein or Creaction protein).ti,ab.
85. 100 or 101
86. Receptors, Interleukin-2/
87. (interleukin-2 receptor$ or interleukin-ii receptor$).ti,ab.
88. (il-2 receptor$ or il-ii receptor$ or il2 receptor$).ti,ab.
89. (sil-2 or sil-2r or sil2 or sil-ii or sil-iir).ti,ab.
90. (t-cell growth factor receptor$ or tcgf receptor$).ti,ab.
91. or/103-107
92. (procalcitonin or pro-calcitonin).ti,ab.
93. calcitonin precursor.ti,ab.
94. 109 or 110
95. Receptors, IgG/
96. igg receptor$.ti,ab.
97. (gamma fc receptor$ or fc gamma receptor$).ti,ab.
98. immunoglobulin g receptor.ti,ab.
99. (leu-11 or leu11).ti,ab.
100. (cdw32 or cd-32 or cd32 or cd-64 or cd64 or cd-16 or cd16).ti,ab.
101. (fc gamma ri or fc gammari or fc gamma rii or fc gammarii or fc gamma riii or fc gammariii).ti,ab.
102. (sfc gamma riii or sfc gammariii).ti,ab.
103. or/112-119
104. Adenosine Deaminase/
105. adenosine deaminase.ti,ab.
106. (ada-1 or ada1 or ada-2 or ada2).ti,ab.
107. (adenosine aminohydrolase or adenosine amino hydrolase).ti,ab.
108. or/121-124
109. Blood Sedimentation/
110. ((erythrocyte or blood) adj sedimentation).ti,ab.
111. 126 or 127
112. Serum Amyloid A Protein/
113. (serum amyloid A or serum amyloid protein a).ti,ab.
114. serum a related protein.ti,ab.
115. amyloid serum protein saa.ti,ab.
116. amyloid-related serum protein.ti,ab.
117. (amyloid a adj (precursor or protein)).ti,ab.
118. (amyloid protein adj (saa or aa)).ti,ab.
119. amyloid fibril protein aa.ti,ab.
120. or/129-136
121. Chemokine CCL2/
122. (monocyte chemotactic protein-1 or monocyte chemoattractant protein-1 or mcp-1).ti,ab.
123. ccl2.ti,ab.
124. or/138-140
125. Neopterin/
126. (neopterin or neopterine).ti,ab.
127. (umanopterin or monapterin).ti,ab.
128. or/142-144
129. lipopolysaccharide-binding protein.ti,ab.
130. lps binding protein.ti,ab.
131. 146 or 147
132. 21 or 24 or 31 or 38 or 50 or 62 or 66 or 72 or 78 or 84 or 87 or 92 or 99 or 102 or 108 or 111 or 120 or 125 or 128 or 137 or 141 or 145 or 148
133. 15 and 147
