# Supplementary material for: Systematic review and meta-analysis of the value of initial biomarkers in predicting adverse outcome in febrile neutropenic episodes in children and young people with cancer
Source: BMC Med. 2012 Jan 18;10:6. doi: 10.1186/1741-7015-10-6 (PMC3331823; doi:10.1186/1741-7015-10-6)
Supplement: Additional file 2 — QUADAS assessments. Full list of assessed QUADAS (diagnostic test accuracy critical appraisal) criteria for the 25 included studies. [file 1741-7015-10-6-S2.DOCX]

**Additional File 2: QUADAS criteria for 25 included studies**

| **Author** | **1** | **2** | **3** | **4** | **5** | **6** | **7** | **8** | **9** | **10** | **11** |
| --- | --- | --- | --- | --- | --- | --- | --- | --- | --- | --- | --- |
| Ammann 2003 | Yes | Yes | Yes | Yes | No | Yes | Yes | Unclear | Unclear | Yes | Yes |
| Barnes 2002 | Yes | No | Yes | Yes | Yes | Yes | Yes | Unclear | Unclear | No | Yes |
| de Bont 1999 | Yes | No | Yes | Yes | Yes | Yes | Yes | Unclear | Unclear | Yes | Yes |
| Diepold 2008 | Yes | No | Yes | Yes | Yes | Yes | Yes | Unclear | Unclear | Yes | Yes |
| Dylewska 2005 a&b | Yes | No | Yes | Yes | Yes | Yes | Yes | Unclear | Unclear | Yes | Yes |
| El-Maghraby 2007 | Unclear | Yes | Yes | Yes | Yes | Yes | Yes | Unclear | Unclear | Yes | Yes |
| Hatzistilianou 2007 | Unclear | No | Yes | Yes | Yes | Yes | Yes | Unclear | Unclear | Yes | Yes |
| Heney 1992 | Yes | Yes | Yes | Yes | Yes | Yes | Yes | Unclear | Unclear | Yes | Yes |
| Hitoglou-Hatzi 2005 | Unclear | No | Yes | Yes | Yes | Yes | Yes | Unclear | Unclear | Unclear | Yes |
| Hodge 2006 | Yes | No | Yes | Yes | Yes | Yes | Yes | Unclear | Unclear | Yes | Yes |
| Katz 1992 | Yes | Yes | Yes | Yes | Yes | Yes | Yes | Unclear | Unclear | Yes | Yes |
| Kitanovski 2006 | Yes | No | Yes | Yes | Yes | Yes | Yes | Unclear | Unclear | Yes | Yes |
| Lehrnbecher 1999 | Yes | No | Yes | Yes | Yes | Yes | Yes | Unclear | Unclear | Yes | Unclear |
| Lehrnbecher 2004 | Yes | No | Yes | Yes | Yes | Yes | No | Unclear | Unclear | Yes | Yes |
| Riikonen 1992 | Yes | Yes | Yes | Yes | Yes | Yes | Yes | Unclear | Unclear | Yes | Yes |
| Riikonen 1993 | Unclear | No | Yes | Yes | Yes | Yes | Yes | Unclear | Unclear | Yes | Yes |
| Santolaya 1994 | Yes | Yes | Yes | Yes | Yes | Yes | Yes | Yes | Yes | Yes | Yes |
| Santolaya 2007 | Yes | Yes | Yes | Yes | Yes | Yes | Yes | Yes | Unclear | Yes | Yes |
| Santolaya 2008 | Yes | Yes | Yes | Yes | Yes | Yes | Yes | Unclear | Yes | Yes | Yes |
| Secmeer 2007 | Unclear | No | Yes | Yes | Yes | Yes | Yes | Unclear | Unclear | Yes | Yes |
| Soker 2001 | Unclear | No | Yes | Yes | Yes | Yes | Yes | Unclear | Unclear | Yes | Yes |
| Spasova 2005 | Yes | No | Yes | Yes | Yes | Yes | Yes | Unclear | Unclear | Yes | Yes |
| Stryjewski 2005 | Yes | Yes | Yes | Yes | Yes | Yes | Yes | Unclear | Unclear | Yes | Yes |
| Santolaya 2001 | Yes | Yes | Yes | Yes | Yes | Yes | Yes | Yes | Yes | Yes | Yes |
| Santolaya 2002 | Yes | Yes | Yes | Yes | Yes | Yes | Yes | Yes | Yes | Yes | Yes |

1 = representative patients, 2 = clearly described selection criteria, 3 = whole sample, or a random selection of sample, received reference standard, 4 = all patients received same reference standard, 5 = index test not part of reference standard, 6 = index test described adequately, 7 = reference standard described adequately, 8 = blinded interpretation of index test results, 9 = blinded interpretation of reference standard results, 10 = same clinical data available as in clinical practice, 11 = adequate reference standard
