# Supplementary material for: Systematic review and meta-analysis of the value of initial biomarkers in predicting adverse outcome in febrile neutropenic episodes in children and young people with cancer
Source: BMC Med. 2012 Jan 18;10:6. doi: 10.1186/1741-7015-10-6 (PMC3331823; doi:10.1186/1741-7015-10-6)
Supplement: Additional file 3 — Per-study results used in meta-analysis. Individual study results used in pooled analyses presented by marker, outcome and cut-off. [file 1741-7015-10-6-S3.DOCX]

**Additional File 3:** Per-study results used in meta-analysis

| **Citation** | **Cutpoint** | **Sensitivity (95% CI)** | **Specificity (95% CI)** | **Method of derivation** |
| --- | --- | --- | --- | --- |
| *CRP: Bacteraemia* | | | | |
| Spasova 2005 | 20 | 1 (95% CI 0.78 to 1) | 0.04 (95% CI 0.01 to 0.18) | mean/sd |
| Spasova 2005 | 50 | 0.21 (95% CI 0.08 to 0.48) | 1 (95% CI 0.88 to 1) | mean/sd |
| Spasova 2005 | 90 | 0 (95% CI 0 to 0.22) | 1 (95% CI 0.88 to 1) | mean/sd |
| Riikonen 1993 | 20 | 0.65 (95% CI 0.41 to 0.83) | 0.3 (95% CI 0.21 to 0.41) | 2*2 extracted from text/graph |
| Riikonen 1993 | 50 | 0.18 (95% CI 0.06 to 0.41) | 0.73 (95% CI 0.62 to 0.82) | 2*2 extracted from text/graph |
| *CRP: Documented & Clinical Infection* | | | | |
| Spasova 2005 | 20 | 1 (95% CI 0.87 to 1) | 0.06 (95% CI 0.01 to 0.28) | mean/sd |
| Spasova 2005 | 50 | 0.92 (95% CI 0.75 to 0.98) | 1 (95% CI 0.81 to 1) | mean/sd |
| Spasova 2005 | 90 | 0.44 (95% CI 0.27 to 0.63) | 1 (95% CI 0.81 to 1) | mean/sd |
| Secmeer 2007 | 50 | 0.68 (95% CI 0.48 to 0.83) | 0.46 (95% CI 0.3 to 0.62) | sensitivity/specificity reported |
| Katz 1992 | 20 | 0.71 (95% CI 0.59 to 0.81) | 0.32 (95% CI 0.22 to 0.44) | sensitivity/specificity reported |
| Katz 1992 | 50 | 0.46 (95% CI 0.34 to 0.58) | 0.75 (95% CI 0.63 to 0.84) | sensitivity/specificity reported |
| Katz 1992 | 100 | 0.22 (95% CI 0.13 to 0.34) | 0.94 (95% CI 0.85 to 0.98) | sensitivity/specificity reported |
| Riikonen 1993 | 20 | 0.62 (95% CI 0.43 to 0.78) | 0.28 (95% CI 0.18 to 0.4) | 2*2 extracted from text/graph |
| Riikonen 1993 | 50 | 0.15 (95% CI 0.06 to 0.34) | 0.71 (95% CI 0.59 to 0.8) | 2*2 extracted from text/graph |
| El-Maghraby 2007 | 90 | 0.69 (95% CI 0.57 to 0.8) | 0.73 (95% CI 0.54 to 0.86) | 2*2 extracted from text/graph |
| Ammann 2003 | 5 | 0.97 (95% CI 0.91 to 0.99) | 0.12 (95% CI 0.08 to 0.18) | sensitivity/specificity reported |
| Ammann 2003 | 50 | 0.48 (95% CI 0.38 to 0.58) | 0.7 (95% CI 0.62 to 0.77) | sensitivity/specificity reported |
| Santolaya 1994 | 40 | 0.95 (95% CI 0.85 to 0.98) | 0.77 (95% CI 0.59 to 0.88) | 2*2 extracted from text/graph |
| Kitanovski 2006 | 60 | 0.63 (95% CI 0.39 to 0.82) | 0.69 (95% CI 0.56 to 0.8) | sensitivity/specificity reported |
| Hitoglou-Hatzi 2005 | 20 | 0.9 (95% CI 0.74 to 0.96) | 0.21 (95% CI 0.11 to 0.36) | mean/sd |
| Hitoglou-Hatzi 2005 | 50 | 0.76 (95% CI 0.58 to 0.88) | 0.74 (95% CI 0.58 to 0.85) | 2*2 extracted from text/graph |
| Hitoglou-Hatzi 2005 | 90 | 0.66 (95% CI 0.47 to 0.8) | 0.87 (95% CI 0.73 to 0.94) | mean/sd |
| Santolaya 2001 | 90 | 0.75 (95% CI 0.69 to 0.81) | 0.8 (95% CI 0.75 to 0.84) | sensitivity/specificity reported |
| Hatzistilianou 2007 | 50 | 0.9 (95% CI 0.8 to 0.95) | 0.79 (95% CI 0.63 to 0.9) | sensitivity/specificity reported |
| *CRP: Gram-ve Bacteramia* | | | | |
| Lehrnbecher 1999 | 20 | 0.88 (95% CI 0.53 to 0.98) | 0.34 (95% CI 0.26 to 0.43) | sensitivity/specificity reported |
| Lehrnbecher 1999 | 50 | 0.88 (95% CI 0.53 to 0.98) | 0.5 (95% CI 0.41 to 0.59) | sensitivity/specificity reported |
| Lehrnbecher 1999 | 100 | 0.88 (95% CI 0.53 to 0.98) | 0.78 (95% CI 0.69 to 0.85) | sensitivity/specificity reported |
| *CRP: Death* | | | | |
| Santolaya 2007 | 90 | 0.79 (95% CI 0.52 to 0.92) | 0.61 (95% CI 0.56 to 0.66) | 2*2 extracted from text/graph |
| *CRP: Sepsis* | | | | |
| Katz 1992 | 20 | 1 (95% CI 0.65 to 1) | 0.32 (95% CI 0.24 to 0.41) | sensitivity/specificity reported |
| Katz 1992 | 50 | 0.71 (95% CI 0.36 to 0.92) | 0.67 (95% CI 0.58 to 0.75) | sensitivity/specificity reported |
| Katz 1992 | 100 | 0.71 (95% CI 0.36 to 0.92) | 0.71 (95% CI 0.62 to 0.79) | sensitivity/specificity reported |
| Santolaya 2008 | 90 | 0.54 (95% CI 0.45 to 0.63) | 0.63 (95% CI 0.59 to 0.67) | 2*2 extracted from text/graph |
| *PCT: Documented & Clinical Infection* | | | | |
| Secmeer 2007 | 0.1 | 0.2 (95% CI 0.09 to 0.39) | 0.74 (95% CI 0.58 to 0.86) | sensitivity/specificity reported |
| Secmeer 2007 | 0.2 | 0.12 (95% CI 0.04 to 0.29) | 0.89 (95% CI 0.74 to 0.95) | sensitivity/specificity reported |
| Secmeer 2007 | 0.3 | 0.11 (95% CI 0.04 to 0.28) | 0.94 (95% CI 0.81 to 0.98) | sensitivity/specificity reported |
| Secmeer 2007 | 0.4 | 0.07 (95% CI 0.02 to 0.23) | 0.94 (95% CI 0.81 to 0.98) | sensitivity/specificity reported |
| Hitoglou-Hatzi 2005 | 0.55 | 0.97 (95% CI 0.83 to 0.99) | 0.58 (95% CI 0.42 to 0.72) | mean/sd |
| Hitoglou-Hatzi 2005 | 0.1 | 0.97 (95% CI 0.83 to 0.99) | 0.47 (95% CI 0.32 to 0.63) | mean/sd |
| Hitoglou-Hatzi 2005 | 0.2 | 0.97 (95% CI 0.83 to 0.99) | 0.5 (95% CI 0.35 to 0.65) | mean/sd |
| Hitoglou-Hatzi 2005 | 0.3 | 0.97 (95% CI 0.83 to 0.99) | 0.5 (95% CI 0.35 to 0.65) | mean/sd |
| Hitoglou-Hatzi 2005 | 0.4 | 0.97 (95% CI 0.83 to 0.99) | 0.53 (95% CI 0.37 to 0.68) | mean/sd |
| Hatzistilianou 2007 | 0.2 | 0.97 (95% CI 0.89 to 0.99) | 0.97 (95% CI 0.85 to 0.99) | sensitivity/specificity reported |
| Kitanovski 2006 | 0.55 | 0.94 (95% CI 0.72 to 0.99) | 0.71 (95% CI 0.58 to 0.82) | sensitivity/specificity reported |
| *PCT: Sepsis* | | | | |
| Santolaya 2008 | 2 | 0.57 (95% CI 0.48 to 0.66) | 0.46 (95% CI 0.41 to 0.5) | mean/sd |
| PCT: Bacteraemia | | | | |
| Kitanovski 2006 | 0.1 | 0.33 (95% CI 0.1 to 0.7) | 0.78 (95% CI 0.65 to 0.87) | sensitivity/specificity reported |
| Kitanovski 2006 | 0.2 | 0.33 (95% CI 0.1 to 0.7) | 0.89 (95% CI 0.79 to 0.95) | sensitivity/specificity reported |
| Kitanovski 2006 | 0.3 | 0.33 (95% CI 0.1 to 0.7) | 0.93 (95% CI 0.83 to 0.97) | sensitivity/specificity reported |
| Kitanovski 2006 | 0.4 | 0.33 (95% CI 0.1 to 0.7) | 0.95 (95% CI 0.86 to 0.98) | sensitivity/specificity reported |
| *IL6: Gram-ve bacteraemia* | | | | |
| Lehrnbecher 1999 | 235 | 1 (95% CI 0.82 to 1) | 0.63 (95% CI 0.53 to 0.72) | sensitivity/specificity reported |
| Lehrnbecher 2000 | 1000 | 0.74 (95% CI 0.51 to 0.88) | 0.96 (95% CI 0.9 to 0.98) | sensitivity/specificity reported |
| *IL6: Documented & Clinical Infection* | | | | |
| Kitanovski 2006 | 235 | 0.88 (95% CI 0.64 to 0.97) | 0.87 (95% CI 0.75 to 0.93) | sensitivity/specificity reported |
| Riikonen 1992 | 235 | 0.1 (95% CI 0.03 to 0.3) | 0.98 (95% CI 0.91 to 1) | 2*2 extracted from text/graph |
| Riikonen 1992 | 1000 | 0 (95% CI 0 to 0.16) | 1 (95% CI 0.94 to 1) | 2*2 extracted from text/graph |
| Lehrnbecher 2004 | 235 | 0.89 (95% CI 0.82 to 0.94) | 0.91 (95% CI 0.86 to 0.94) | sensitivity/specificity reported |
| Lehrnbecher 2004 | 1000 | 0.11 (95% CI 0.06 to 0.18) | 0.99 (95% CI 0.97 to 1) | sensitivity/specificity reported |
| Diepold 2008 | 42 | 0.9 (95% CI 0.81 to 0.94) | 0.86 (95% CI 0.69 to 0.94) | sensitivity/specificity reported |
| *IL6: Bacteraemia* | | | | |
| Diepold 2008 | 240 | 0.64 (95% CI 0.39 to 0.84) | 0.75 (95% CI 0.65 to 0.82) | sensitivity/specificity reported |
